# ARCS: Active Radar Cross Section for Multi-Radiator Problems in Complex EM Environments

**DOI:** 10.3390/s20123371

**Published:** 2020-06-14

**Authors:** Liqiang Niu, Yongjun Xie, Peiyu Wu, Chungang Zhang

**Affiliations:** School of Electronic and Information Engineering, Beihang University, Beijing 100191, China; liqiangniu@buaa.edu.cn (L.N.); yjxie@buaa.edu.cn (Y.X.); cgzhang@buaa.edu.cn (C.Z.)

**Keywords:** active radar cross section (ARCS), complex electromagnetics environment, coherent characteristics, external disturbance, multi-radiators

## Abstract

In order to analyze the scattering properties in multi-radiator problems, the active radar cross section (ARCS) concept is proposed under complex electromagnetic (EM) environments. The corresponding calculation methods and formulation are proposed by incorporating the monostatic radar cross section (RCS) concept with external disturbances. By introducing the phase characteristics into the ARCS concept, the coherent problems can be accurately solved. Through analyzing the external disturbance and the radar waves by employing the finite element method, the coherent and the incoherent characteristics of the external disturbance can be simulated in complex structures. Numerical examples and an experiment are carried out to further demonstrate the effectiveness of the proposed ARCS concept. The results demonstrate that the proposed ARCS concept obtains better universality compared with the existing incoherent multi-radiator formulation. Meanwhile, the ARCS can be identical with the solution which is obtained by the single radar wave. Compared with the existing incoherent methods for external disturbances calculations, the proposed ARCS concept is more rational. Through the experiment, the effectiveness of the calculation method and formulation is further demonstrated and validated.

## 1. Introduction

With the development of electronic devices and radar systems, the electromagnetic (EM) environment becomes more increasingly complex than ever before [1,2,3]. In complex and various EM environments, the analysis of scattering problems, the improvement of radar systems and the recognition of hidden targets have become frontier science in recent years [4,5,6,7], including electronic countermeasures, electronic support and so on [8,9]. When solving scattering problems, the investigation of the scattering properties is regarded as one of the most important aspects [8,9,10]. Especially in complex EM environments, the scattering properties will be affected by the incidence of multi-radiators [11]. Hence, the investigation of target scattering properties in complex EM environments has been established as of great value both for military and civil employments [12,13].

Within various parameters, the radar cross section (RCS) is regarded as one of the most important ones during the analyzing and predicting of scattering property problems [14,15]. The radar is a device for transmitting and receiving electromagnetic waves for the purpose of accurately detecting and identifying the target. The RCS of the target is usually measured by radar. The radar is often calibrated to make data accurate before it is used. Active radar calibrators are used to calibrate the radar [16,17,18]. Thus, RCS has been extensively employed and investigated by various countries and experts [19,20,21]. Different full-wave analysis methodologies have been introduced during RCS investigation, such as the method of moment (MoM) [22,23,24] and finite difference time domain (FDTD) [25,26,27,28]. In recent decades, high-frequency approximate methods have been introduced, such as geometrical optics [29], physical optical (PO) [30,31], geometric diffraction [32,33], consistent diffraction [34], equivalent current [35,36] and physical diffraction (PD) [37,38]. In recent years, to not only decrease the computational duration but also reduce the computational consumption, several recurrence methods and fast methods have been carried out. The conjugate gradient fast Fourier transform method has been introduced to solve the linear equations which are formed in the MoM method [39,40]. The fast multipole method and the multilevel fast multipole algorithm have been introduced to improve the computational efficiency by increasing the speed of the multiplicity between the matrices and vectors [41,42,43,44]. As one of the most preventative methodologies in solving Maxwell’s equations, the finite element method (FEM), proposed by R. Courant in 1943, has received much attention in solving RCS problems [45,46,47]. With its advantages of considerable accuracy and wide applications, the FEM has received much attention [48,49]. 

With the development of computational technology, the modeling of high-accuracy three-dimensional structures has become easier to implement. A series of software has been proposed to analyze, calculate and predict RCS in complex structures [50,51]. The RCS software combines the structure modeling with the RCS calculation through the friendly human–computer interface. By this means, a series of software has been introduced, for example, XPATCH, CADDSCAT, RESPECT, RANURS, GRECO and HFSS [52,53,54,55,56,57]. Based on the shooting bouncing ray technique, the XPATCH can calculate the EM scattering in complex structures by employing different implementations [52]. Based on the common object request broker architecture algorithm, various functions can be extended by the XPATCH. Based on the PO and PD methods, the computational efficiency graphical electromagnetic computing method has been introduced to simulate the RCS in the high-frequency band. The simulation results obtained by GRECO can be converted into visual graphics, which makes them easier to analyze [56]. With the advantages of high reliability, short computational duration and mature grid generation, HFSS shows its considerable potential in analyzing EM scattering problems among complex structures. Recently, HFSS has received much attention in analyzing RCS problems [58,59,60].

However, the above-mentioned high-frequency approximate methods and full-wave methods are mainly focused on the efficiency of the RCS calculation. The comprehensive effect between multi-radiators and radar waves has a huge influence on resultants in complex EM environments. Especially when the radar waves and the multi-radiators have the same frequencies and phases, the coherent characteristics have not been simulated in previous work. The insignificant influence will result in unacceptable computational rationality. The effects, together with the contribution from the radar waves and the multi-radiators, makes the modulation effect different from that of single radar problems. Meanwhile, the cheating, distribution and destroying of the targets are no longer to be neglected among series of frequency devices. The influence of RCS on multi-radiators has attracted much attention. To the best of our knowledge, no studies have investigated the simulation methods in the coherent characteristics of external disturbances. Previous works mainly investigated the incoherent characteristics of external disturbances. The simulation of scattering properties in complex EM environments, especially the analyzing and the modeling of the incidence of multi-radiators and the coherent external disturbances, has become a frontier science.

Here, the active RCS (ARCS) concept and the ARCS calculation formulation are proposed to analyze the complex structures in complex EM environments. By incorporating the radar equations and the proposed formulations, the coherent and incoherent characteristics of external disturbances can be calculated. Numerical examples and an experiment including the metal cylinder structure, the prismatic model, the F22 model and the active cancellation structure are carried out to demonstrate the uniformity, the effectiveness and the application of the proposed formulation and concept. The results demonstrate that the proposed ARCS concept obtains better universality compared with previous work. Not only the coherent and incoherent characteristics but also the statistical characteristics of external disturbances are demonstrated. Through the experiment, the application of the proposed ARCS concept can be further illustrated. It is indicated in the results that the proposed ACRS shows its advantage in analyzing coherent problems for complex structures in complex EM environments, especially active cancellation, EM stealth and so on. 

## 2. Concept Demonstration

The conventional RCS concept, which was introduced in [21], represents the ratio between the power of incident waves and total waves under the isotropic scattering situation. The definition of conventional RCS can be given as

(1) $$\sigma =\underset{R\to \infty }{\lim}4\pi R^{2}\frac{{\left|E_{s}\right|}^{2}}{{\left|E_{i}\right|}^{2}}$$

where $\sigma \;\left(m^{2}\right)$ is RCS, $E_{s}\left(V/m\right)$ is the scattering field of receiving antenna in the radar system, $E_{i}\left(V/m\right)$ is the incident field and R (m) is the distance between radar and target. When R is an infinitely large value, the distance between the radar and the antenna satisfies the far-field condition. Thus, the influence of the distance on RCS can be eliminated.

**Figure 1:**
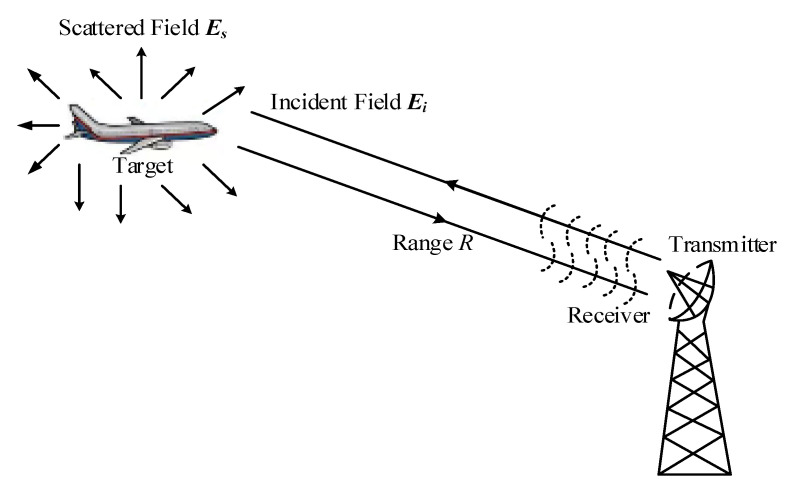
A sketch picture of the radar cross section (RCS) concept.

As shown in Figure 1, the scattering ability can be reflected by the RCS value. When the target is incident by the radar, the power received by the target is scattered in various directions. Thus, the power distribution becomes different in space. The power distribution is defined as the scattering field which can be reflected by RCS. In view of induced current, the scattering field comes from the second radiation of induced current on the surface of the target.

RCS is a complex physical quantity which is related to the geometry parameters and physical parameters, such as size, shape and material; the parameters of incidence waves, such as frequency, polarization direction and waveform; and the attitude angle between the radar and the targets.

**Figure 2:**
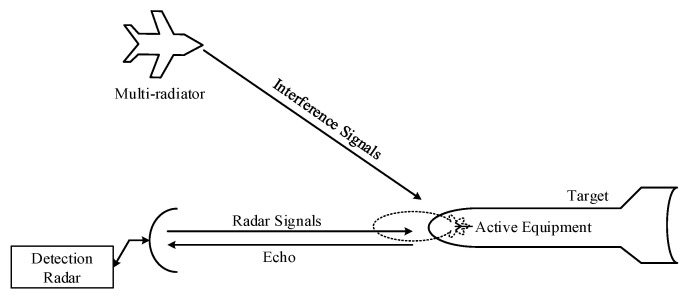
The sketch picture of the multi-radiators problem.

However, the conventional RCS concept is only efficient in single radiator problems. When the target is incident by the multi-radiators, the scattering properties of targets change compared with the single radiator situation. The reason is that the waves which come from the multi-radiators and the radar wave work together, resulting in the occupation of modulation effect. Meanwhile, the scattering effect is generated between the radiated waves of the active equipment on the target and the scattering echo waves, resulting in the scattering waves diversification. The sketch picture of the multi-radiators problem is shown in Figure 2.

The scattering echo waves are caused by the radar, multi-radiators and active equipment working together. The radar outside the target, the radiators outside the target and the frequency equipment on the surface of the target work together towards the target, resulting in the second radiation in space. This second radiation is called ARCS. Here, the definition of ARCS can be proposed as

(2) $$\sigma^{\prime }=\underset{R\to \infty }{\lim}4\pi R^{2}\frac{{\left|E_{s}\right|}^{2}}{{\left|E_{iR}\right|}^{2}}$$

where $\sigma^{\prime }\;\left(m^{2}\right)$ denotes the ARCS, $E_{iR}\;\left(V/m\right)$ is the incident field which is radiated on the target by radar and R (m) is the distance between radar and target. Similar to the RCS, the impact of distance on ARCS can be eliminated when R is infinitely large. $E_{s}\;\left(V/m\right)$ is the scattering field of the receiving antenna in the radar system under the multi-radiators, which can be calculated as

(3) $$E_{s}=E_{s\_iR}+E_{s\_iM}$$

where $E_{s\_iR}$ is the scattering field caused by $E_{iR}$, $E_{s\_iM}$ is the total field which can be obtained by adding the $E_{iM}$ with the waves from surface active equipment and $E_{iM}$ is the electric field radiated by multi-radiators.

**Figure 3:**
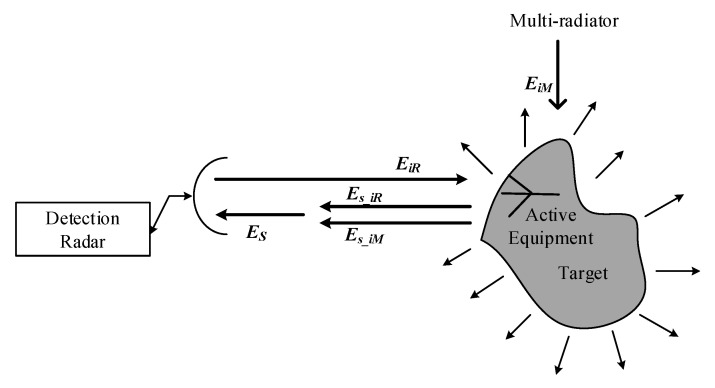
The sketch picture of the active radar cross section (ARCS) concept.

The sketch picture of the ARCS concept is shown in Figure 3. It can be observed that $E_{s}$ of ARCS is composed of the radar, multi-radiators and active equipment working together on the target. Meanwhile, it can be concluded that $E_{s}$ of ARCS is completely different compared with RCS.

ARCS can not only be affected by geometry parameters, physical parameters, incidence wave parameters and attitude angle but also by multi-radiators and frequency equipment. The detection, tracking and reorganization of the target will be affected by the multi-radiators and the frequency equipment, resulting in the increment of false-alarm probability. In conclusion, the simulation and calculation of ARCS, especially phase polarization mode, incidence wave intensity and multi-radiators and frequency equipment, are of vital importance.

## 3. Formulation

### 3.1. Calculation and Simulation of ARCS

**Figure 4:**
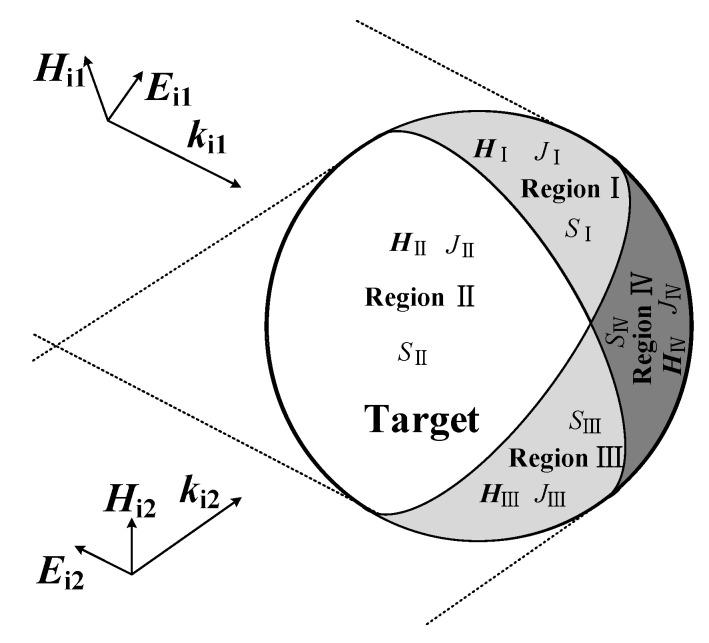
An arbitrary object illuminated by two plane waves.

Consider an arbitrary object illuminated by two plane waves ($k_{i1}$ and $k_{i2}$ are wave vectors and $E_{i1}$, $H_{i1}$ and $E_{i2}$, $H_{i2}$ are electric and magnetic fields of two waves) as shown in Figure 4. S is assumed to represent a closed surface of the target. The far zone scattering fields of the conductive target can be expressed as [38]

(4) $$E_{S}\left(r^{\prime }\right)=-j\omega A\left(r^{\prime }\right)-\nabla \Phi \left(r^{\prime }\right),\;\;\;\;r^{\prime }\in S$$

where $A\left(r^{\prime }\right)$ and $\Phi \left(r^{\prime }\right)$ are vector and scalar potentials occurred by the surface current at S, ω is the radar frequency and $r^{\prime }$ is the position vector on S.

Surface S can be divided into four parts. Region I can be expressed as $S_{I}$, which is illuminated by incident plane wave ($E_{i1}$, $H_{i1}$); region II, expressed as $S_{\mathrm{II}}$, is illuminated by two incident plane waves ($E_{i1}$, $H_{i1}$ and $E_{i2}$, $H_{i2}$); region III is illuminated by incident plane wave ($E_{i2}$, $H_{i2}$), and region IV is not illuminated. Surface S can be expressed as

(5) $$S=S_{I}+S_{\mathrm{II}}+S_{\mathrm{III}}+S_{\mathrm{IV}}$$

Assuming $E_{iI}$, $E_{\mathrm{iII}}$, $E_{\mathrm{iIII}}$ and $H_{iI}$, $H_{\mathrm{iII}}$, $H_{\mathrm{iIII}}$ represent the incident electric and magnetic field, respectively, of $S_{I}$, $S_{\mathrm{II}}$ and $S_{\mathrm{III}}$, one obtains

(6a) $$E_{iI}=E_{i1}$$

(6b) $$E_{\mathrm{iII}}=E_{i1}+E_{i2}$$

(6c) $$E_{\mathrm{iIII}}=E_{i2}$$

(7a) $$H_{iI}=H_{i1}$$

(7b) $$H_{\mathrm{iII}}=H_{i1}+H_{i2}$$

(7c) $$H_{\mathrm{iIII}}=H_{i2}$$

In order to solve scattering fields of perfectly E condition (PEC) targets, the Green’s function $G_{A}\left(r,r^{\prime }\right)$ was introduced. The vector and the scalar potentials can be expressed as [38]

(8) $$A\left(r\right)=\mu \int_{S}G_{A}\left(r,r^{\prime }\right)\cdot J\left(r^{\prime }\right)dS^{\prime }$$

(9) $$\nabla \Phi \left(r\right)=-\frac{1}{\varepsilon }\int_{S}\rho \left(r^{\prime }\right)\cdot \nabla G_{A}\left(r,r^{\prime }\right)dS^{\prime }$$

where A(r) and Φ(r) are the vector and scalar potentials at r, r is the position vectors for observation point, $dS^{\prime }$ is the increment area, μ and ε are the permeability and permittivity, $\rho \left(r^{\prime }\right)$ is the charge density and $J\left(r^{\prime }\right)$ is the surface current density. Assuming $J_{I}$ , $J_{\mathrm{II}}$ , $J_{\mathrm{III}}$, $J_{\mathrm{IV}}$, $H_{sI}$, $H_{s\mathrm{II}}$, $H_{s\mathrm{III}}$, $H_{s\mathrm{IV}}$ and $H_{I}$, $H_{\mathrm{II}}$, $H_{\mathrm{III}}$, $H_{\mathrm{IV}}$ represent currents, scattering magnetic fields $H_{sn}\left(r^{\prime }\right)$ and total magnetic fields $J_{n}\left(r^{\prime }\right)$, respectively, of $S_{I}$, $S_{\mathrm{II}}$,$S_{\mathrm{III}}$ and $S_{\mathrm{IV}}$, one obtains

(10) $$H_{sn}\left(r^{\prime }\right)=H_{n}\left(r^{\prime }\right)-H_{i}{}_{n}\left(r^{\prime }\right),\;\;\;r^{\prime }\in S_{n},\;n\in I,\;\mathrm{II},\;\mathrm{III},\;\mathrm{IV}$$

(11) $$J_{n}\left(r^{\prime }\right)=\hat{n}\times H_{n}\left(r^{\prime }\right)$$

where $\hat{n}$ is the unit normal outward from the surface. By substituting (7)–(11) into (4), the far zone (r) scattering field $E_{S}\left(r\right)$ can be expressed as

(12) $$E_{S}\left(r\right)=-j\omega \mu \sum_{n=I}^{\mathrm{IV}}\int_{S_{n}}G_{A}\left(r,r^{\prime }\right)\cdot J_{n}\left(r^{\prime }\right)dS^{\prime }-\frac{1}{\varepsilon }\sum_{n=I}^{\mathrm{IV}}\int_{S_{n}}\rho \left(r^{\prime }\right)\cdot \nabla G_{A}\left(r,r^{\prime }\right)dS^{\prime },\;\;\;\;\;r^{\prime }\in S_{n}$$

As can be observed, the multi-radiator wave can change the magnetic field on the target according to (11). Thus, the scattering field can be changed according to (12). The far electric field can be expressed as

(13) rE=r⋅E

where rE is the far electric field, which can be obtained by multiplying E with the corresponding r, r is the radiation radius and E is the electric field at the distance of r. Generally, rE can be regarded as the scattering field, and one obtains

(14) $$E_{s}=\frac{{\left(rE\right)}_{s}}{R}$$

where ${\left(rE\right)}_{s}$ is the scattering field of rE. By substituting (14) into (2), the ARCS can be written in the final form as

(15) $$\sigma^{\prime }=4\pi \frac{{\left|{\left(rE\right)}_{s}\right|}^{2}}{{\left|E_{s\_iR}\right|}^{2}}$$

### 3.2. Coherent and Incoherent Calculation of ARCS

**Figure 5:**
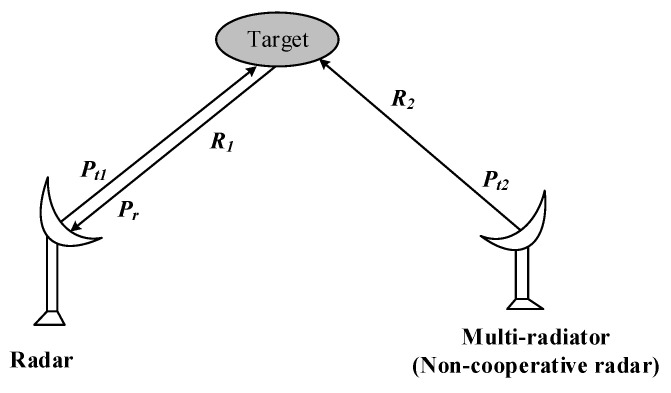
The incoherent and coherent situations of the external disturbances.

The impact of radar echo can be analyzed through the coherent and incoherent characteristics. In the incoherent situation, the received radar power of scattering waves is the addition of scattering power, which is generated by the incidence of radar and the incidence of multi-radiators, as shown in Figure 5.

The incoherent external disturbance can be calculated as

(16) $$P_{r}^{}=\frac{P_{t1}G_{1}^{2}\lambda_{1}^{2}\sigma_{1}}{{\left(4\pi \right)}^{3}R_{1}^{4}}+\frac{P_{t2}G_{1}G_{2}\lambda_{2}^{2}\sigma_{2}}{{\left(4\pi \right)}^{3}R_{1}^{2}R_{2}^{2}}$$

where $P_{t1}$, $G_{1}$, $\lambda_{1}$, $R_{1}$ and $\sigma_{1}$ are radar radiated power, radar gain, radar wavelength and distance between radar and target and the monostatic RCS. $P_{t2}$, $G_{2}$, $\lambda_{2}$, $R_{2}$ and $\sigma_{2}$ are the multi-radiator radiated power, multi-radiator gain, multi-radiator wavelength, distance between multi-radiator and target and bistatic RCS, respectively. The incoherent external disturbance can be obtained by employing (15) and (16) as

(17) $$\sigma^{\prime }=4\pi \frac{{\left|{\left(rE\right)}_{s}^{M}\right|}^{2}+{\left|{\left(rE\right)}_{s}^{R}\right|}^{2}}{{\left|E_{iR}\right|}^{2}}$$

where ${\left(rE\right)}_{s}^{M}$ is the scattering field directed to the radar when $E_{iM}$ incidents the object separately and ${\left(rE\right)}_{s}^{R}$ is the backward scattering field when $E_{iR}$ incidents the object separately. However, Equation (17) is no longer efficient in the calculation of total radar receiving power under coherent situations. The reason is that the stable relationship is formed between the radar and multi-radiators. To alleviate such a problem, the synthetic electric field on the surface of the target is calculated as follows

(18) $$\left|E_{i}\right|=E_{iR}e^{j\varphi_{1}}+E_{iM}e^{j\varphi_{2}}$$

where $\varphi_{1}$ and $\varphi_{2}$ are the phase of radar wave and the phase multi-radiators, respectively, and $E_{i}$ is the synthetic electric field. The power of echo wave which is received by the radar can be given as

(19) $$P_{r}^{}=\frac{{\left|E_{i}\right|}^{2}\lambda^{2}G_{1}\sigma^{\prime }}{2\eta {\left(4\pi \right)}^{2}R_{1}^{2}}$$

where λ is the wavelength of the EM waves and η is the wave impedance in a vacuum.

### 3.3. Active Cancellation Technology

In [61,62,63], the interference effect is analyzed from the perspective of active cancellation. The interference effect will be generated with the incidence of the same frequency of multiple waves, resulting in the addition and the cancellation of the field amplitude. More precisely, the phenomenon is caused by the strengthened and weakened wave vibration. The active cancellation can be described from several aspects as follows: 1) active electronic equipment, which is loaded on the target body emits waves to interfere with the incoming wave enemy; and 2) scattering field, which is weakened along the direction which can be detected by the enemy radar.

Supposing that the RCS at radar and cancellation signal are $\sigma_{0}$ and $\sigma_{1}$, respectively, the phases of the radar incident wave and the cancellation wave are $\varphi_{0}$ and $\varphi_{1}$. RCS aiming at consultant wave can be given as

(20) $$\sigma ={\left|\sqrt{\sigma_{0}}e^{j\varphi_{0}}+\sqrt{\sigma_{1}}e^{j\varphi_{1}}\right|}^{2}$$

Equation (20) can be further obtained as

(21) $$\sigma =\sigma_{0}\left|1+\frac{\sigma_{1}}{\sigma_{0}}+2\sqrt{\frac{\sigma_{1}}{\sigma_{0}}}\cos\left(\varphi_{1}-\varphi_{0}\right)\right|$$

## 4. Numerical Results, Experiment Results And Discussion

To demonstrate the ARCS concept for multi-radiator problems, numerical examples and an experiment are introduced, respectively. Firstly, a metal cylinder structure is employed to demonstrate the unity of the proposed ARCS and conventional RCS. Secondly, the coherent theory is introduced in the ARCS concept. Thirdly, the ARCS statistics characteristic is investigated through the F22 model. Finally, the active cancellation based on the ARCS concept is simulated and tested experimentally. Here, the numerical examples are simulated by HFSS.

### 4.1. Unity of ARCS and RCS

In order to further demonstrate the ARCS concept in different applications, it is necessary to validate the uniformity between the ARCS and RCS. In this section, the ARCS concept is testified through a metal cylinder structure with a single incidence wave. The unity is illustrated through comparison.

**Figure 6:**
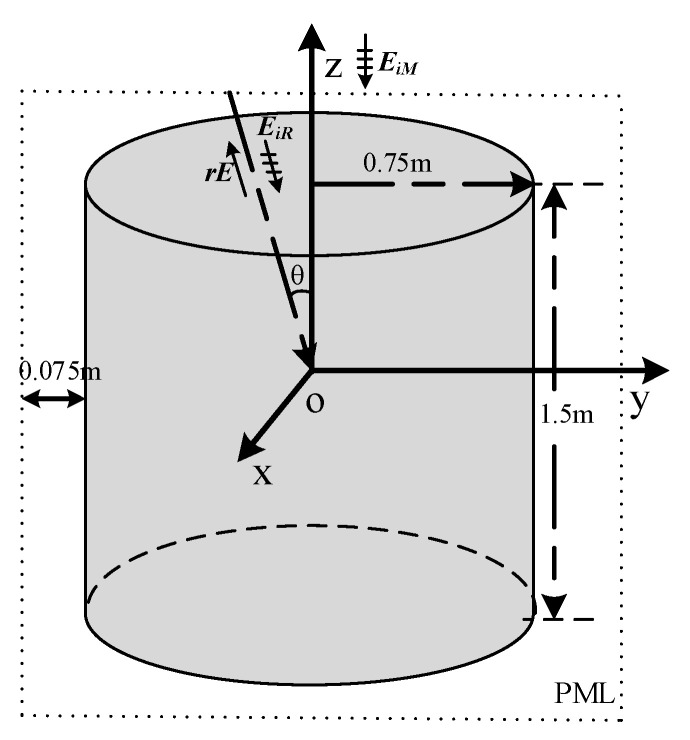
The model of the metal cylinder structure in the comparison of RCS and ARCS.

As shown in Figure 6, the incidence wave is a plane wave with a maximum frequency of 1 GHz and wavelength (λ) of 0.3 m. The wave $E_{iR}$ in the xOz plane incidents along the negative side of the z-direction. The polarization direction is along the y-direction. In this calculation, $E_{iM}=0$ is chosen to validate the uniformity. rE is the scatted field by the target. The metal cylinder structure has a radius of 0.75 m (2.5λ) and a height of (5λ). The metal material can be simulated by employing the perfect E conductor (PEC). The perfectly matched layer (PML) is employed at boundaries. The distances between the object and the boundaries are 0.075 m. In this simulation, rE is observed for further calculation.

**Figure 7:**
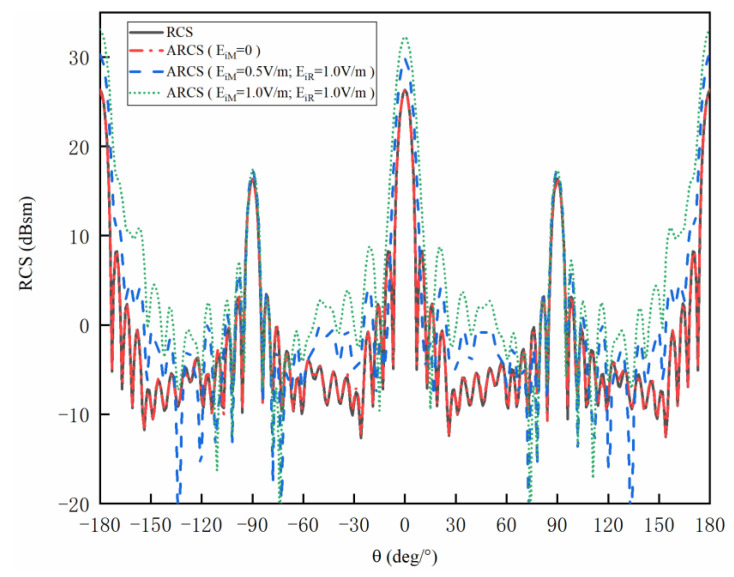
The RCS and ARCS with different $E_{iM}$ of the metal cylinder structure.

Figure 7 shows the RCS and ARCS versus different angles and external disturbances of the metal cylinder structure. It can be observed that the curves are almost overlapped when $E_{iM}=0$, indicating that the ARCS with the single incident wave holds the same accuracy as RCS. The maximum values are 26.32 dBsm and 16.28 dBsm, with the angles of 0° and 90°, respectively. The reason is that mirror reflection plays an important role among the backward scattering when EM waves incident vertically to the structure. The error between the RCS and ARCS is 0.1 dBsm when the angle is 90°. With the whole angles band, the average error between ARCS and RCS is 0.07 dBsm. Thus, the ARCS and RCS show considerable uniformity. Meanwhile, ARCS increases with the increment of $E_{iM}$. The most obvious situations can be found at 0. The ARCS are 26.34 dBsm, 29.78 dBsm and 32.28 dBsm with $E_{iM}$ of 0 V/m, 0.5 V/m and 1 V/m, respectively.

### 4.2. Comparison of ARCS between the Coherent Distribution and Incoherent Distribution

**Figure 8:**
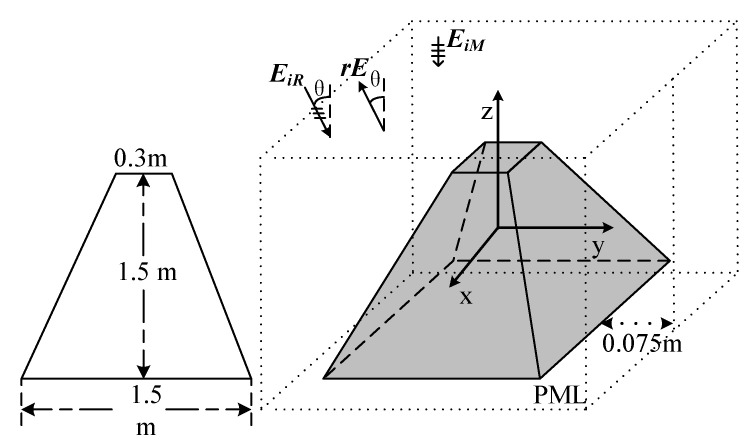
The model of the prismatic in the demonstration of the coherent and incoherent characteristics of ARCS.

To further demonstrate the characteristics of the coherent and incoherent distribution at different phases, a prismatic model is carried out. As shown in Figure 8, the top and bottom of the PEC prismatic model are 0.3 m and 1.5 m, respectively. The height of the model is 1.5 m. The incidence wave $E_{iR}$ which is polarized along the y-direction, incidents from the xOz plane with the range between −180° to 180°. The maximum frequency of $E_{iR}$ is 1 GHz.

The directions of the multi-radiators’ electronic fields $E_{iM}$ which have the same polarization direction with $E_{iR}$ are assumed as the opposite of the z-direction. The boundaries conditions and the position of the model are the same as the numerical example above. To obtain the ARCS, the scattering field rE is observed.

**Figure 9:**
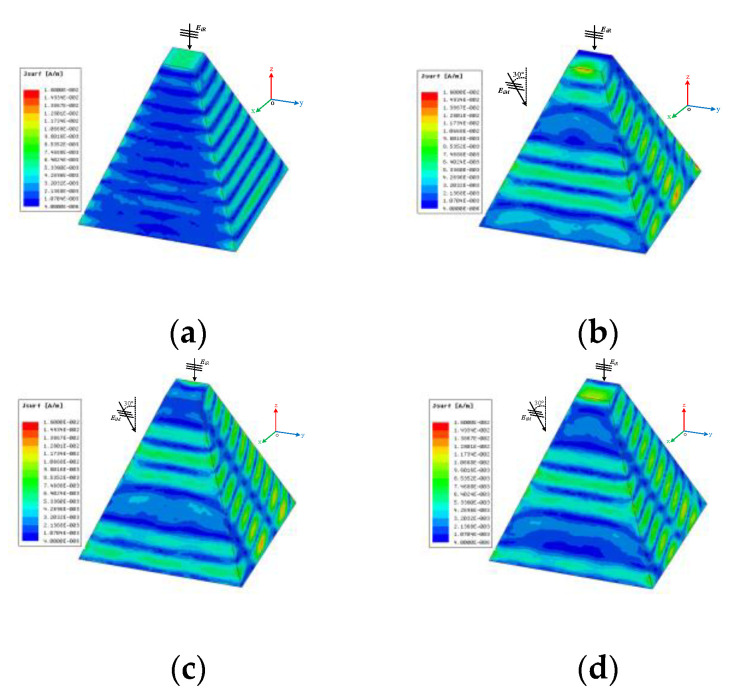
The surface current of the metal cylinder under different incident waves. (**a**) Single incident by $E_{iR}$ $E_{iR}$**,** $E_{iM}$ with the phase difference of 0° (**c**) $E_{iR}$**,** $E_{iM}$ with the phase difference of 180° (**d**) $E_{iR}$**,** $E_{iM}$ with the Phase Difference of 90°.

As shown in Figure 9, when the object is incident by the radar wave and the multi-radiators, the surface-induced current of the metal prismoid model is different compared with a single radar wave. The radar wave is incident along the *z*-axis. The multi-radiator wave from 30° is with the same power and polarization direction with the incident wave. Figure 9a shows the current distribution of the surface of metal prismoid with the incidence of the radar wave. Figure 9b shows the surface current distribution of the surface of the metal prismoid model with the incidence of both the radar wave and the multi-radiator wave. The phase difference between the radar wave and multi-radiator wave is 0°. Figure 9c shows the surface-induced current distribution of the surface of the metal prismoid model with a different phase angle of 180°. Figure 9d shows the surface-induced current distribution of the surface of the metal prismoid model with a different phase angle of 90°.

**Figure 10:**
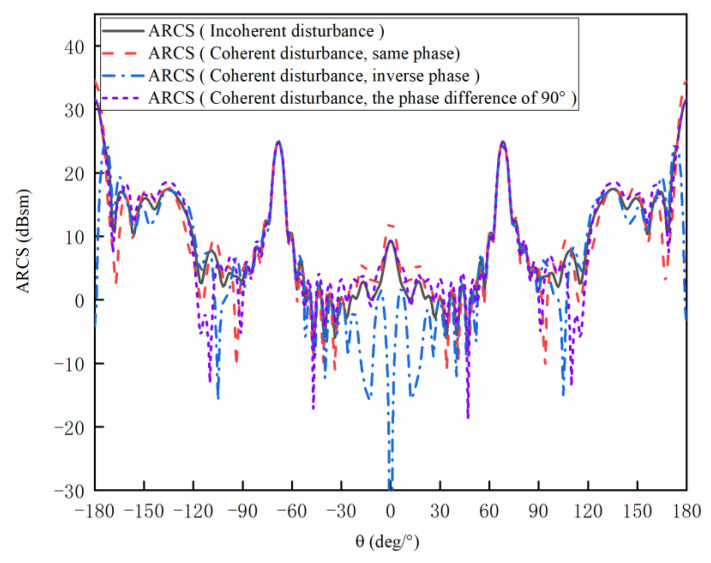
Coherent external disturbances and incoherent external disturbances of the prismatic model.

Figure 10 shows the coherent external disturbances with the same, inverse and 90° difference in phases and incoherent external disturbances in (16) [64]. It can be observed that the curves are symmetrical at 0°. The reason is that the prismatic model is a symmetrical structure. Thus, the characteristics at the right side of 0° are discussed here. The left side can be obtained by employing the same approach.

When the incidence wave and the multi-radiator wave hold the same phases, the coherent external disturbance ARCS is 3.01 dBsm higher than the incoherent at 0°. From 0° to 25° and 175° to 180°, the coherent external disturbances are 2.00 dBsm higher than the incoherent external disturbances. Within the whole degree band, the maximum difference between the coherent and incoherent external disturbance is 8.93 dBsm. From 25° to 55° and 90° to 175°, a large difference occurs. Between 110° to 175°, the coherent external disturbances and incoherent external disturbances are almost overlapped.

Comparing the coherent external disturbances at the inverse phase with the incoherent external disturbances, it can be calculated that the ARCS of coherent external disturbance is 319.32 dBsm lower than that of the incoherent external disturbance at 0°. From 110° to 175°, the curves are almost overlapped. At the other angles, a large calculation difference can be observed.

Comparing the coherent external disturbances at the phase difference of 90° with the incoherent external disturbances, it can be observed that the ARCS of coherent external disturbance is almost overlapped with that of the incoherent external disturbance around an angle of 0°.

As can be concluded from the second numerical example, the difference between the coherent external disturbances and incoherent external disturbances is substantial. The reason is that the phase of the electric field is not considered during the calculation of incoherent external disturbances. When echoes of backward scattering fields from radar and multi-radiator hold the same polarization directions and phases, the backward scattering of the total field can be significantly enhanced. Meanwhile, the backward total scattering field will be reduced when they hold the same polarization directions and inverse phases. In conclusion, the coherent external disturbances play an important part in the analysis of the scattering properties.

### 4.3. The Statistical Characteristics of ARCS

**Figure 11:**
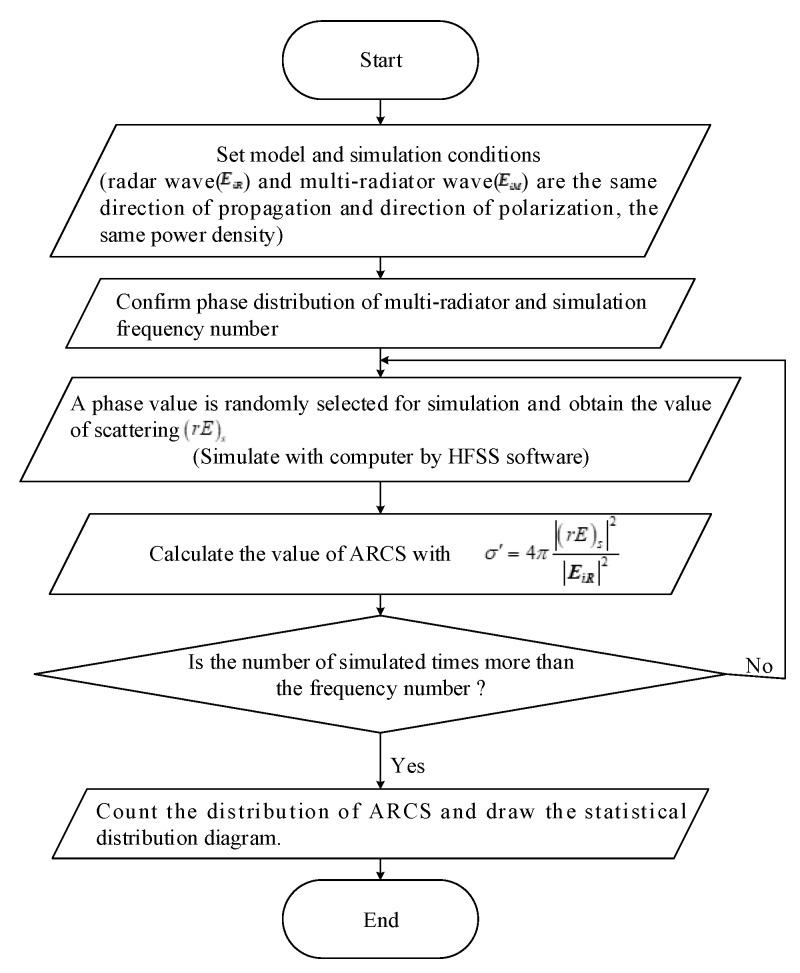
The diagram of the statistic ARCS by a random phase of multi-radiators.

In the complex EM environment, the phase randomness of the multi-radiator has a huge influence on the scattering properties, especially in the calculation of RCS. To investigate such a phenomenon, the statistical electromagnetics theory is employed. The phase of the multi-radiator is considered as a stochastic variable. By this means, the ARCS and the statistic characteristics can be analyzed. Figure 11 shows the diagram of the processing during the analysis of the statistical characteristics.

It can be observed that the processing can be divided into three parts for demonstration as follows:

(1) The model, simulation conditions, confirm multi-radiator phase distribution and frequency number are set.

(2) The phase value is selected to simulate the ${\left(rE\right)}_{s}$ by HFSS under certain frequency numbers.

(3) The distribution of ARCS is counted to obtain the final solution.

**Figure 12:**
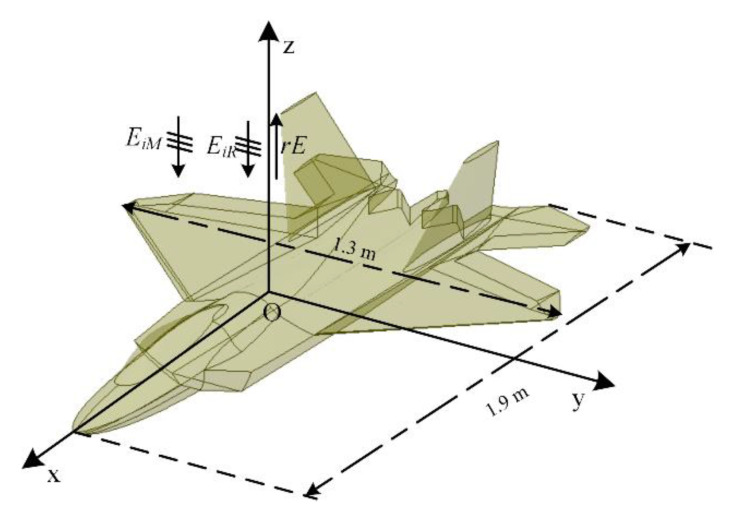
The sketch picture of the metal F22 structure.

Figure 12 shows the model of an F22 [65]. The PEC F22 has dimensions of 1.9 × 1.3 m in the x- and y-directions. More details can be found in [65]. The parameters of the incidence wave and the boundary conditions are the same as the numerical examples above. $E_{iR}$ and $E_{iM}$ are incident along the negative side of z-direction. The polarization direction of $E_{iM}$ and $E_{iR}$ are along the y-direction. To obtain the statistical characteristics of ARCS, the scattering field rE is observed along the z-direction.

**Figure 13:**
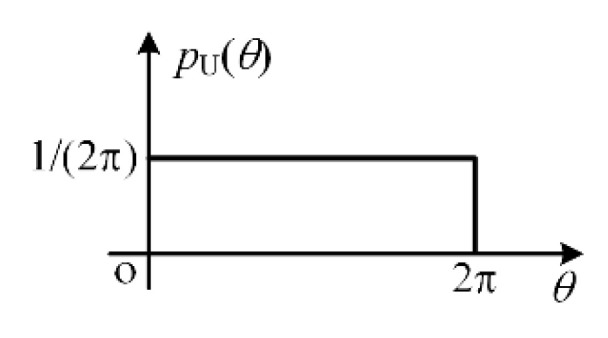
The uniform distribution of the multi-radiator phase.

In this simulation, the phases of the multi-radiator with different probability distributions are considered. Firstly, the uniform distribution of [0, 2π] is considered, which can be given as

(22) $$p_{U}\left(\theta \right)=\left\{ \begin{array}{l} \frac{1}{2\pi },\;0\leq \theta \leq 2\pi \\ 0,\;others \end{array}\right.$$

where $p_{U}\left(\theta \right)$ is the probability density of the uniform distribution. To further illustrate the ARCS with different phases, multi-radiators with the uniform distribution are simulated randomly 500 times. Figure 13 shows the uniform distribution of the multi-radiator in this simulation.

**Figure 14:**
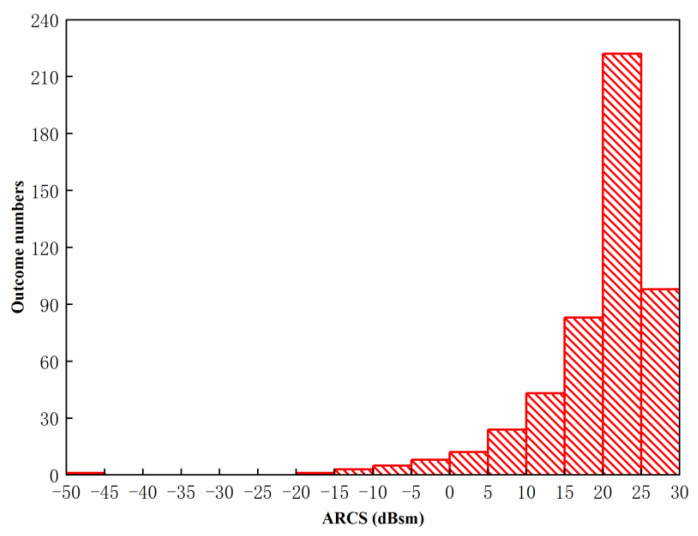
Statistical characteristics of ARCS obtained by the uniform distribution multi-radiators with 500 times random simulation.

Figure 14 shows the statistical characteristics of ARCS obtained by the uniform distribution multi-radiators with 500 times random simulation. The ARCS range, the outcome frequencies and the outcome rate are shown in Table 1. It can be observed from Table 1 and Figure 14 that the maximum outcome of ARCS has a number of 222 and a proportion of 44.40%, which occurred between 20 dBsm to 25 dBsm. It concluded that the outcomes of ARCS improve with the increment of ARCS value from −50 dBsm to 25 dBsm. Ranging from 15 dBsm to 30 dBsm, the ARCS outcomes are with the most proportion of 80.60%.

**Figure 15:**
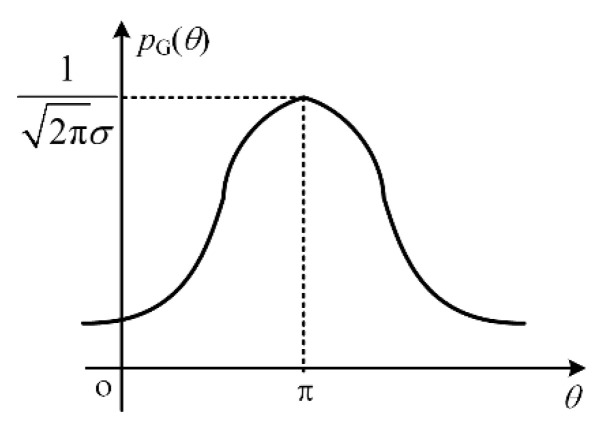
The Gaussian distribution of the multi-radiator phase.

The Gaussian distribution of [0,2π] with the average value of μ=π and standard deviation of σ=0.05 is considered. The probability density can be given as

(23) $$p_{N}\left(\theta \right)=\left\{ \begin{array}{l} \frac{1}{\sqrt{2\pi }\sigma }e^{-\frac{{\left(\theta -\mu \right)}^{2}}{2\sigma^{2}}},\;0\leq \theta \leq 2\pi \\ 0,\;others \end{array}\right.$$

where θ∼N(μ,σ) is the probability density of the Gaussian distribution. Meanwhile, ARCS, which is influenced by the multi-radiator with the Gaussian distribution, is calculated randomly 500 times. The Gaussian distribution of multi-radiator is shown in Figure 15.

**Figure 16:**
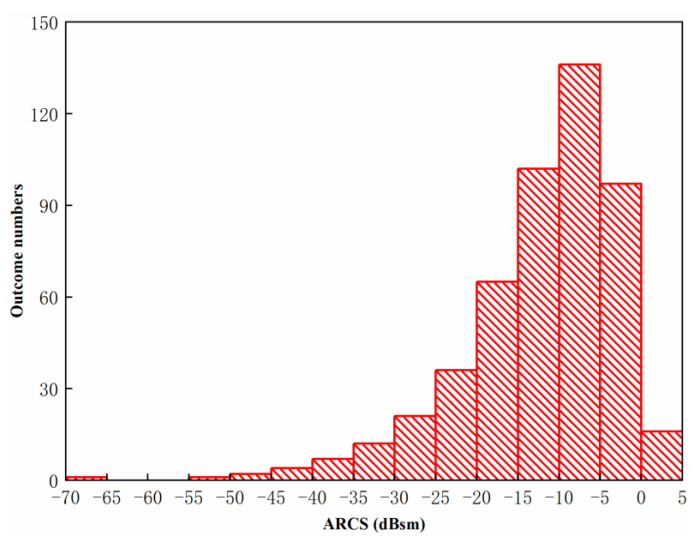
Statistic characteristics of ARCS obtained by the Gaussian distribution multi-radiators with 500 times random simulation.

It can be established that most values are concentrated near the average point π. Thus, the probability of the phase with the value of [π−0.15,π+0.15] can be calculated as 99.74%. The statistic characteristics of ARCS obtained by the Gaussian distribution are shown in Figure 16. As shown, the distribution of ARCS follows Gaussian distribution. Table 2 shows the ARCS range, the outcome frequencies and the outcome rate at the Gaussian distribution. As shown from Figure 16 and Table 2, the outcome rates of ARCS with the range from −20 dBsm to −10 dBsm and −10 dBsm to 0 dBsm are 33.40% and 46.60%, respectively. The outcome numbers under the multi-radiator phase with Gaussian distribution decreases with the decrement of ARCS. As is concluded from both Figure 16 and Table 2, the outcome numbers account for 80.00%, with the ARCS from −20 dBsm to 0 dBsm.

### 4.4. ARCS Concept Applied to the Active Cancellation

The ARCS concept can be applied to the active cancellation. Through the active cancellation, the scattering intensity will be reduced and its stealth performance will be improved at the same time. To investigate the application of ARCS, the interaction between the wave of the antenna on the surface of the target and the echo of radar are considered. For the scattering of the target containing the antenna, the influence of the antenna structure and mode scatterings are considered.

**Figure 17:**
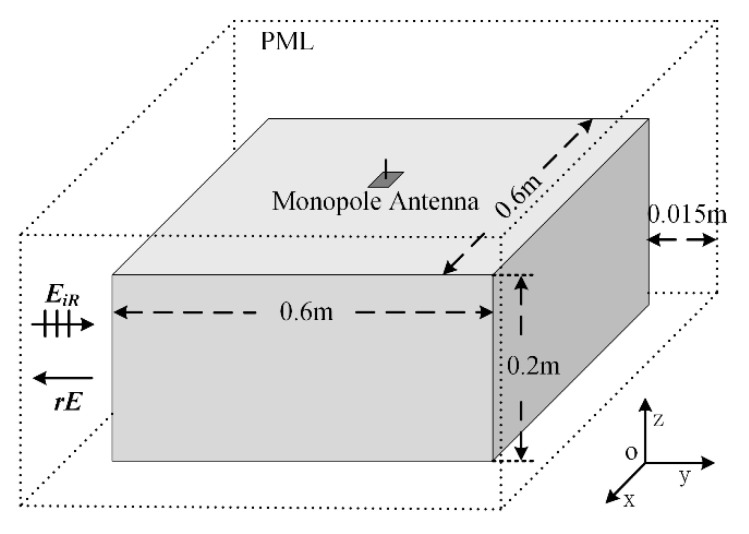
The sketch picture of active cancellation structure.

In this section, a numerical example is introduced. The effectiveness of the proposal is validated through the experiment. As shown in Figure 17, the PEC metal cuboid structure has dimensions of 0.6 m × 0.6 m × 0.2 m. A monopole antenna with a length of 0.05 m in the z-direction is located at the center of the top surface. $E_{iR}$ is the incident plane wave with the maximum frequency of 5 GHz. $E_{iR}$, which has the polarization direction along the z-direction, incidents from the positive y-direction. The distances between the cuboid structure and the boundaries are 0.015 m. The PML is employed to absorb the outgoing waves. ARCS is calculated through rE along the negative sides of the y-direction. In this simulation, the antenna is excited by the inverter phase excitation with -10 dBm. The ACRS is calculated through the comparison of active cancellation. When the antenna is excited, the active cancellation can be obtained.

From the simulation results, it can be observed that the ARCS with the active cancellation is 2.83 dBsm lower than that without active cancellation. This indicates that the wave scattering can be decreased by employing the scattering waves which interact with the multi-radiators and the external radar with the same frequency and inverse phase.

**Figure 18:**
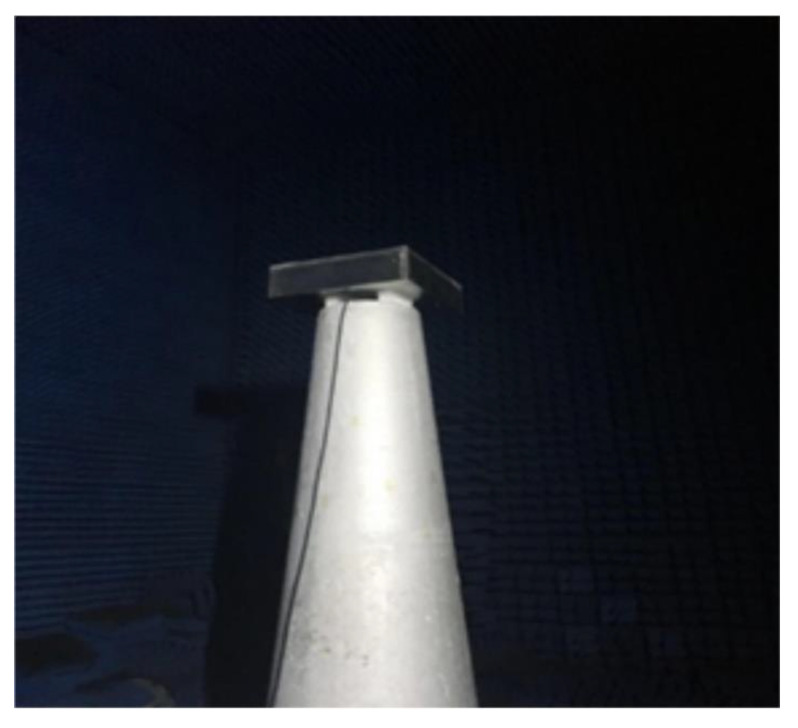
The experimental condition in the microwave dark chamber.

To further investigate the effectiveness of the proposal, an experiment is introduced in the microwave dark chamber. Figure 18 shows a photograph of the experiment. The object is the same as that in the numerical example above. The metal cuboid structure is made up of aluminum. To simulate the plane wave, the compact field with 5 GHz is incident. On the surface of the metal cuboid structure, a monopole antenna with a length of 0.05 m is located at the center. The antenna is excited by the −10 dBm source with the inverse phase and the same frequency compared with the incidence wave. Table 3 shows the results obtained by the simulation and experiment with different conditions. As shown in the experiment, ARCS is 0.9 dBsm lower than that of the simulation without the active cancellation. Furthermore, the experiment is 0.13 dBsm lower than that of the simulation with the active cancellation. This indicates the multi-radiators affect the scattering properties.

## 5. Conclusions

Here, the ARCS concept and formulation are proposed to analyze the problems in complex EM environments. Through numerical examples and an experiment, the uniformity, effectiveness, characteristics and application are demonstrated and tested. The coherent and incoherent characteristics of ARCS are illustrated through the scattering properties with multi-radiators. The results indicate that the ARCS concept and formulations can be degenerated to monostatic situations. Furthermore, the stochastic phase characteristic is investigated through a F22 model. The ARCS statistical characteristics are given through different distributions, indicating the relationship between the ARCS and the different phases. Meanwhile, the application of ARCS is illustrated by employing the active cancellation experiment. All in all, the ARCS for multi-radiators can be regarded as a simplified method for the calculation of target scattering properties in the complex EM environments, especially active cancellation, EM stealth and so on.

## Figures and Tables

**Table 1:** The ARCS range, the outcome frequencies and the outcome rate at the uniform distribution.

ARCS Range (dBsm)	Outcome Frequency (Times)	Outcome Rate (%)
−50~−45	1	0.20
−45~−20	0	0.00
−20~−15	1	0.20
−15~−10	3	0.60
−10~−5	5	1.00
−5~0	8	1.60
0~5	12	2.40
5~10	24	4.80
10~15	43	8.60
15~20	83	16.60
20~25	222	44.40
25~30	98	19.60

**Table 2:** The ARCS range, the outcome frequencies and the outcome rate at the Gaussian distribution.

**ARCS Range** **(dBsm)**	**Outcome Frequency** **(Times)**	**Outcome Rate** **(%)**
−70~−65	1	0.20
−65~−55	0	0.00
−55~−50	1	0.20
−50~−45	2	0.40
−45~−40	4	0.80
−40~−35	7	1.40
−35~−30	12	2.40
−30~−25	21	4.20
−25~−20	36	7.20
−20~−15	65	13.00
−15~−10	102	20.40
−10~−5	136	27.20
−5~0	97	19.40
0~5	16	3.20

**Table 3:** The results obtained by the simulation and experiments with different conditions.

Conditions	Simulation ARCS (dBsm)	Experimental ARCS (dBsm)	Differ (%)
Antenna excited (Active cancellation)	14.59	14.46	0.89
Antenna unexcited (Without Active cancellation)	17.42	16.52	5.10
